# NAADP Signaling: New Kids on the Block

**DOI:** 10.3390/cells11061054

**Published:** 2022-03-21

**Authors:** Andreas H. Guse

**Affiliations:** The Calcium Signaling Group, Department of Biochemistry and Molecular Cell Biology, University Medical Centre Hamburg-Eppendorf, 20246 Hamburg, Germany; guse@uke.de

**Keywords:** NAADP, Ca^2+^ signaling, Ca^2+^ microdomains, dual NADPH oxidase (DUOX), hematological and neurological expressed 1-like protein (HN1L)/Jupiter microtubule-associated homolog 2 (JPT2), ryanodine receptor (RYR), two-pore channel (TPC)

## Abstract

Nicotinic acid adenine dinucleotide phosphate (NAADP) is a universal Ca^2+^ mobilizing second messenger essential for initiation of Ca^2+^ signaling. Recently, novel molecular mechanisms of both its rapid formation upon receptor stimulation and its mode of action were discovered. Dual NADPH oxidase 2 (DUOX2) and hematological and neurological expressed 1-like protein (HN1L)/Jupiter microtubule-associated homolog 2 (JPT2) were discovered as NAADP-forming enzyme and NAADP receptor/binding protein—the new kids on the block. These novel aspects are reviewed and integrated into the previous view of NAADP signaling.

## 1. Introduction

Nicotinic acid adenine dinucleotide phosphate (NAADP) was first described in 1995 by Hon Cheung Lee’s group as highly potent Ca^2+^ releasing second messenger in sea urchin egg homogenates [1]. Soon afterwards, NAADP was also shown to evoke Ca^2+^ release in different cell types from higher eukaryotes [2,3,4], demonstrating its universal role as Ca^2+^ mobilizing second messenger.

## 2. NAADP Formation by NADPH Oxidases (NOX) or Dual NADPH Oxidases (DUOX)

NAADP was initially discovered as an impurity in commercial NADP preparations [5]. The first biosynthetic pathway described was catalysis of NAADP from NADP and nicotinic acid via the so-called “base-exchange reaction” [6]. Here, nicotinamide is completely replaced by nicotinic acid through the multi-functional enzyme NAD-glycohydrolase/ADP-ribosyl cyclase CD38 or ADP-ribosyl cyclase from *Aplysia californica* [6]. Although this mechanism appears straightforward, it requires acidic pH (pH 4–5), thereby limiting the cellular site for efficient NAADP generation to the lumen of acidic endo-lysosomal compartments.

**Figure 1:**
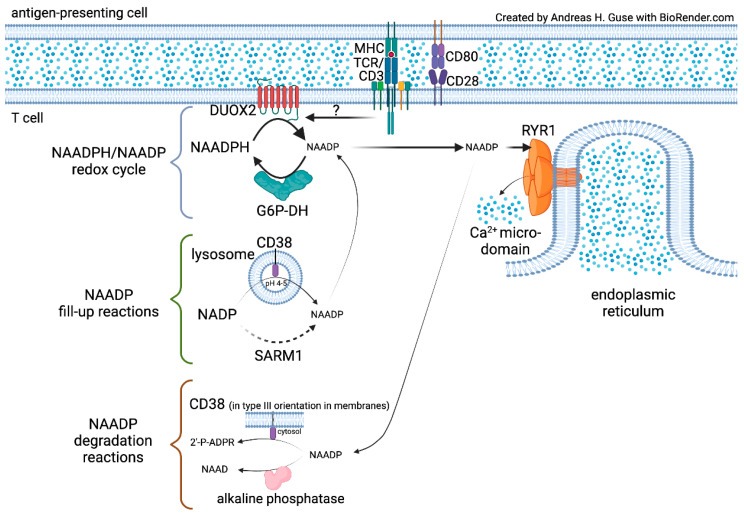
NAADPH/NAADP redox cycle and fill-up and degradation reactions.

Recently, we described a novel pathway for rapid formation and degradation of NAADP by a redox cycle involving NADPH oxidases (NOX) or dual NADPH oxidases (DUOX) for generation of NAADP from its reduced form, NAADPH (Figure 1) [7]. The backward reaction is catalyzed by glucose-6-phosphate dehydrogenase (G6P-DH) (Figure 1). This novel principle of an NAADPH/NAADP redox cycle was analyzed in membranes from HEK293 cells overexpressing NOX5. This isozyme was used as a model enzyme, since its overexpression requires just transfection of a single polypeptide chain, without the need to co-transfect several other subunits. In enzyme kinetics, NAADPH was a similarly well-accepted substrate for NOX5 as compared to NADPH; the pH optimum for NAADPH was even closer to cytosolic conditions as for NADPH [7]. Additionally, the dual NADPH oxidases—DUOX1 and DUOX2, produce NAADP from NAADPH under physiological conditions [7]. Formation of NAADP from NAADPH was observed by wildtype T cell membranes indicating a role for NOX/DUOX family enzymes in NAADP formation. However, since T cells express different NOX and DUOX isozymes, one major task was the identification of the enzyme(s) involved in NAADP formation upon TCR/CD3 stimulation (see below Section 3).

The backward reaction of the NAADPH/NAADP redox cycle was only catalyzed by G6P-DH, but not by other major dehydrogenases tested (Figure 1) [7]. However, we cannot exclude that other NADP-dependent dehydrogenases are also able to produce NAADPH from NAADP. In contrast to NOX 5, the kinetic properties of G6P-DH towards NAADP vs. NADP were quite different—the affinity was about 19-fold higher for NAADP, while the maximum velocity was much smaller for NAADP as compared to NADP [7]. Nevertheless, the low nanomolar concentrations detected in T cell extracts [8] fit to these kinetic parameters (see discussion section of [7]). 

The newly discovered redox cycle of NAADPH/NAADP provides a smart possibility to rapidly produce and degrade NAADP to trigger initial Ca^2+^ signals. In principle, interconversion of NAADPH into NAADP, and vice versa, may work without substantial de *novo synthesis* of either nucleotide. In reality, it is likely that some of the NAADP is converted by degrading reactions to either 2′-phospho-ADPR (2′-P-ADPR) by CD38 in type III orientation, or to nicotinic acid adenine dinucleotide (NAAD) by alkaline phosphatase [9] (Figure 1). Recently, we demonstrated that a very small amount of CD38 is actually found in type III orientation in T cells [10] and a knock-out of *Cd38* results in higher endogenous NAADP concentrations in lymphoid tissues as compared to wildtype tissue [11]. Therefore, the NAADPH/NAADP redox cycle likely requires fill-up reactions as a house-keeping function. A candidate for such fill-up reaction starting from NADP is the “base-exchange reaction” that is catalyzed by both CD38 and sterile α and Toll/interleukin-1 receptor (TIR) motif-containing 1 (SARM1) [12]. In fact, it was recently demonstrated that NAADP can be produced in the lysosomal lumen, where the acidic pH allows for the “base-exchange reaction” [13] (Figure 1). SARM1, though structurally unrelated to CD38, also requires an acidic pH for the “base-exchange reaction” [12]; however, localization in the lumen of endo-lysosomes of SARM1′s active site has not yet been described.

## 3. Search for the NAADP Forming NOX/DUOX Isozyme in T Cells

When it became clear that NOX/DUOX family NADPH oxidases also catalyze oxidation of NAADPH, the question of which isozyme(s) might actually produce NAADPH in living T cells upon T cell receptor/CD3 stimulation arose. T cells express high amounts of NOX1 and NOX2, and much smaller amounts of DUOX1 and DUOX2 [7]. T cells from *Nox1^−/−^*, *Nox2^−/−^*, or *Duoxa1^−/−^/Duoxa2*^−/−^ mice were analyzed for global Ca^2+^ signaling as read-outs since deletion of the NAADP receptor/binding protein HN1L/JPT2 had resulted in characteristic partial attenuation of global Ca^2+^ signaling [14]. Surprisingly, deletion of the highly expressed *Nox1* or *Nox2* did not affect global Ca^2+^ signaling, whereas combined functional deletion of both DUOX1 and DUOX2 by knock-out of the genes of their maturation factors DUOXA1 and DUOXA2, resulted in a similar characteristic decrease of global Ca^2+^ signaling, e.g., delayed signal onset and partially decreased peak and plateau Ca^2+^ signaling [7]. As expected, T cells from *Duoxa1^−/−^/Duoxa2*^−/−^ mice showed reduced numbers of Ca^2+^ microdomains in the first 25s following TCR/CD3 ligation [7]. Selective knock-out of *Duox1^−/−^* or *Duoxa2*^−/−^ in T cells resulted in differential effects—while absence of DUOX2 massively reduced the number of Ca^2+^ microdomains in the first approximately 10s, no effect of deletion of *Duox1^−/−^* was observed suggesting that DUOX2 produces NAADP in the first seconds of T cell activation [7].

## 4. Ca^2+^ Mobilization by NAADP

Some years after the discovery of NAADP’s Ca^2+^ mobilizing activity [1,2,3,4], different NAADP-sensitive Ca^2+^ channels were proposed as follows: type 1 ryanodine receptor (RYR1) [15,16] and two-pore channels (TPC) [17,18,19,20]. For ligand-gated ion channels, there are several examples for direct binding of small molecular ligands to the channels, e.g., d-*myo*-inositol 1,4,5-trisphosphate receptor [21], also suggesting that NAADP might directly bind to these target channels. However, when using a NAADP photoaffinity label in different mammalian cell types, specific labelling of small cytosolic proteins instead of large, membrane-bound Ca^2+^ channels was observed in 2012 [22,23]. Based on this somewhat unexpected discovery, I published a “unifying hypothesis” proposing that NAADP’s Ca^2+^ mobilizing activity via either RYR1 or TPCs may be explained by a specific receptor/binding protein that upon binding to NAADP activates either Ca^2+^ channel type [24]. However, it took another nine years until identification of these small cytosolic NAADP receptors/binding proteins was accomplished; hematological and neurological expressed 1-like protein (HN1L)/Jupiter microtubule-associated homolog 2 (JPT2) was identified by the Jonathan Marchants group and my own laboratory, and published back-to-back in *Science Signaling* [14,25].

Interestingly, a structurally unrelated protein, Lsm12, was also identified as NAADP receptor/binding protein [26]. Lsm proteins are known to bind RNA and are involved in regulation of gene expression, e.g., by facilitating degradation or modification of RNA. Lsm12 was reported to couple NAADP signaling to activation of TPC2 [26].

**Figure 2:**
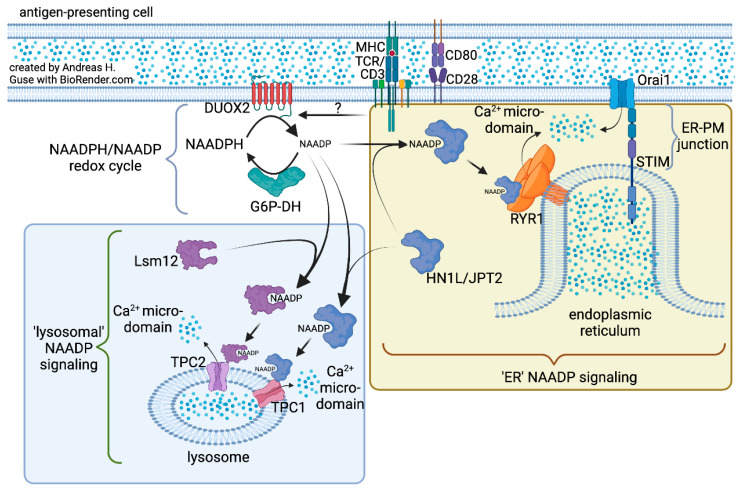
Ca^2+^ mobilization by NAADP mediated by NAADP receptors/binding proteins linking to different organelles and ion channels.

Figure 2 summarizes our current knowledge regarding mechanisms underlying NAADP-evoked Ca^2+^ signaling. The “ER NAADP signaling” model was built mainly from experimental data obtained in T cells. Here, NAADP binds to HN1L/JPT2 that is either localized in the cytosol or already pre-bound to RYR1 [14]. This interaction activates RYR1 resulting in local Ca^2+^ microdomains [14,27] that are almost instantaneously amplified by Ca^2+^ entry processes, store-operated Ca^2+^ entry (Figure 2) [28], and ATP-dependent activation of purinergic P2X4 and P2X7 receptors [29]. RYR1 were localized by super-resolution microscopy in very close proximity to Orai1 channels in the plasma membrane (PM) [28], suggesting that ER–PM junctions are the points of origin of NAADP-evoked Ca^2+^ microdomains. Recent mathematical modeling of this process revealed that RYR1 is likely not directly localized within the ER–PM junction, but somewhat more distant, e.g., below the tip of ER–PM junctions (as schematically drawn in Figure 2) [30]. Despite ample experimental evidence in T cells, the “ER NAADP signaling model” has not been widely accepted, since results in other cell types indicate involvement of endo-lysosomes. This situation was recently discussed in detail [31] and will not be repeated here.

In contrast to direct activation of RYR1 by NAADP bound to HN1L/JPT2, experimental evidence from other cell systems favors the involvement of lysosomes as organellar targets and TPCs as target Ca^2+^ channels (Figure 2, “lysosomal” NAADP signaling). While HN1L/JPT interacts with TPC1 [24], Lsm12 binds to TPC2 [25]. Thus, activation of TPC1 and TPC2 appears to be individually regulated by the two different NAADP receptors/binding proteins (Figure 2). As for the “ER NAADP signaling model”, the role of TPCs as Ca^2+^ channels has also been questioned—Haoxing Xu and colleagues demonstrated that TPCs are Na^+^ channels that are regulated by phosphatidylinositol 3,5-bisphosphate, but not by NAADP [32]. As above, this controversial situation was recently discussed [31] and will not be repeated in detail here. Of note, Christian Grimm and colleagues showed in a paradigm shifting study that the ion selectivity of TPC2 can be tuned to either Na^+^ currents or Ca^2+^ currents, depending on the ligand used, whereas NAADP or the synthetic TPC2-A1-N compound evoked Ca^2+^ currents, Na^+^ currents were stimulated by phosphatidylinositol 3,5-bisphosphate or synthetic TPC2-A1-P [33].

In summary, the newly discovered proteins, the “new kids on the block”, fill important gaps within the NAADP/Ca^2+^ signaling pathway. Especially, the NAADP receptors/binding proteins will allow to further dissect NAADP’s molecular mechanism of action in different cell types and/or under different stimulation conditions.

## Data Availability

Not applicable.
